# Overall and quality-specific plant-based dietary patterns and all-cause mortality in older adults with cardiovascular disease: a prospective cohort study from the CLHLS

**DOI:** 10.3389/fnut.2026.1899346

**Published:** 2026-09-09

**Authors:** Xiaomo Yang, Ruixue Xiao, Yongjun Wu, Hui Chen, Qiuping Zhao

**Affiliations:** 1 Fuwai Central China Cardiovascular Hospital, Central China Subcenter of the National Center for Cardiovascular Diseases, Zhengzhou, Henan, China; 2 Henan University of Chinese Medicine, Zhengzhou, China; 3 Zhengzhou University, College of Public Health, Zhengzhou, China

**Keywords:** all-cause mortality, cardiovascular disease, CLHLS, hPDI, older adults, PDI, plant-based diet, uPDI

## Abstract

**Aims:**

To examine the associations of overall plant-based diet index (PDI), healthy plant-based diet index (hPDI), and unhealthy plant-based diet index (uPDI) with all-cause mortality among older Chinese adults with cardiovascular disease using data from the China Longitudinal Healthy Longevity Survey (CLHLS).

**Methods and results:**

We analyzed 1,526 participants aged 65 years or older from the 2008 wave of the CLHLS cardiovascular disease population, with mortality follow-up through 2019 (heart disease without stroke: *n* = 856; cerebrovascular disease without heart disease: *n* = 480; both: *n* = 190). Dietary intake was assessed using a food frequency questionnaire and converted into PDI, hPDI, and uPDI. Cox proportional hazards models estimated hazard ratios (HRs) and 95% confidence intervals (CIs) for all-cause mortality. The proportional hazards assumption was evaluated using Schoenfeld residuals, and dose-response relationships were examined using restricted cubic splines.

**Conclusion:**

Among older Chinese adults with cardiovascular disease, greater adherence to an overall plant-based dietary pattern was associated with lower all-cause mortality, whereas greater adherence to an unhealthy plant-based dietary pattern was associated with higher mortality.

## Introduction

Cardiovascular disease (CVD) remains a leading cause of premature death and disability worldwide, and the burden is particularly pronounced among older adults (1, 2). Although secondary prevention has traditionally focused on pharmacological therapy and risk-factor control, dietary patterns are increasingly recognized as potentially relevant to long-term prognosis in patients with cardiovascular disease. For older adults living with established CVD, dietary patterns may influence survival through multiple pathways, including blood pressure regulation, lipid metabolism, glycaemic control, systemic inflammation, endothelial function, oxidative stress, and nutritional resilience (3–5).

Plant-based diets have received increasing attention in cardiovascular prevention. However, the term plant-based diet does not necessarily indicate a healthy dietary pattern (6, 7). A diet rich in whole grains, vegetables, fruits, legumes, nuts, and tea may have different implications from a plant-based pattern dominated by refined grains, sugar-rich foods, and high-sodium preserved vegetables. To address this heterogeneity, the overall plant-based diet index (PDI), healthy plant-based diet index (hPDI), and unhealthy plant-based diet index (uPDI) have been developed to distinguish plant-food quantity from plant-food quality.

Existing studies have suggested that higher adherence to healthy plant-based dietary patterns is associated with lower risks of cardiometabolic disease and mortality, whereas unhealthy plant-based patterns may be associated with higher risk (8–10). Nevertheless, evidence among older Chinese adults with prevalent cardiovascular disease remains limited. This population is clinically important because older adults with CVD often have high absolute mortality risk, multiple comorbidities, altered nutritional needs, and dietary structures that differ from Western populations. In China, traditional dietary patterns include both healthy plant foods and potentially deleterious plant-derived foods such as refined grains and preserved vegetables, making quality-specific evaluation especially relevant (11).

Therefore, this prospective cohort study aimed to examine the associations of PDI, hPDI, and uPDI with all-cause mortality among older adults with cardiovascular disease in China. We further explored whether the associations were consistent across demographic and lifestyle subgroups, whether dose-response relationships were approximately linear, and whether the findings were robust in sensitivity analyses, including analyses using standardized scores and plant-only dietary scores.

## Methods

### Study design and population

**Figure 1:**
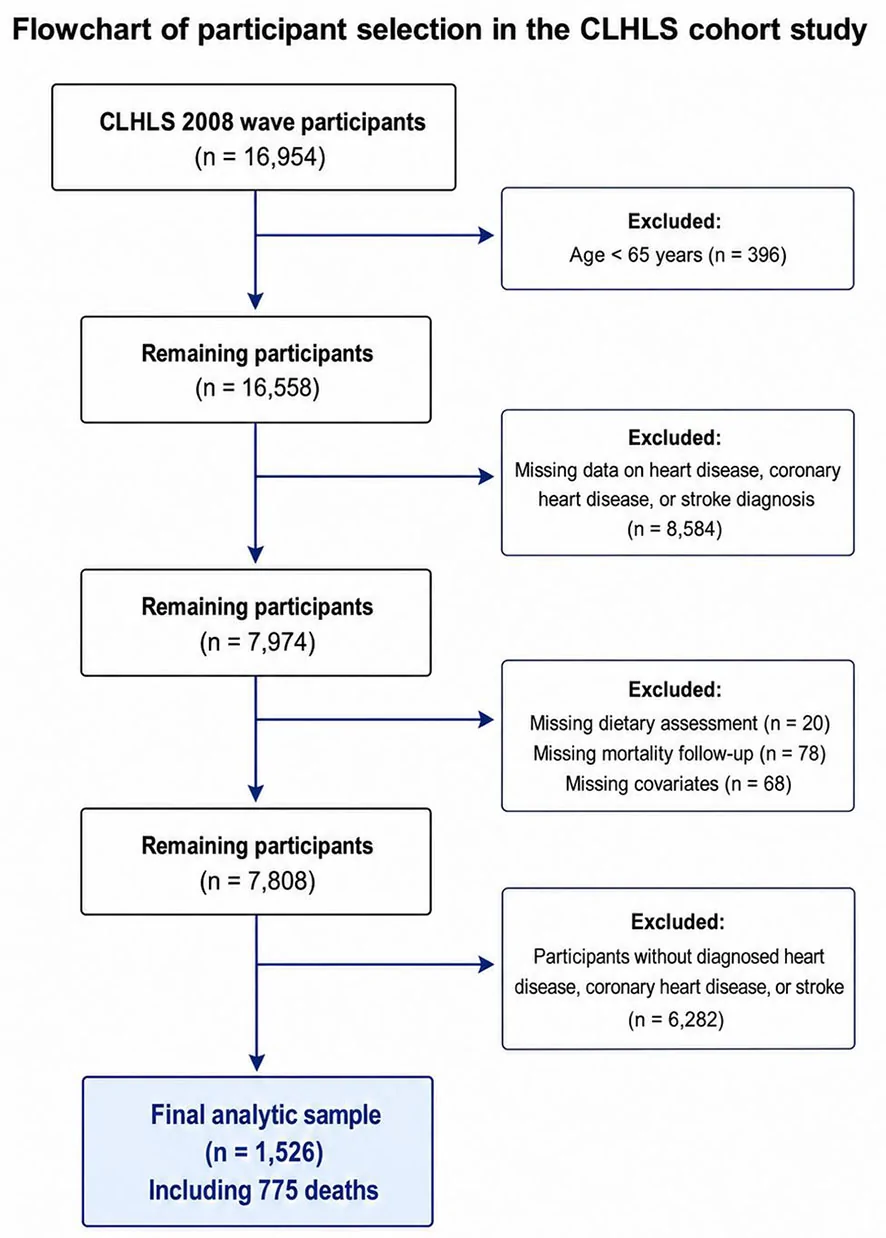
Flowchart.

From the 2008 wave CLHLS, there were 16,558 participants aged ≥65 years. Of these, 8,584 had missing information on CVD diagnostic variables and were excluded first. Among the remaining 7,974 participants, 1,526 with prevalent CVD were included in the final analytic sample after excluding those with missing dietary data (*n* = 20), missing key covariates (*n* = 68), or missing survival status (*n* = 78) (Figure 1).

In the CLHLS, reasons for missing cardiovascular disease diagnostic information include proxy respondent interviews (where certain health conditions could not be reliably ascertained), cognitive impairment limiting self-report accuracy, and the use of abbreviated questionnaires in certain survey waves or sub-samples. These factors contribute to incomplete CVD ascertainment and represent a potential source of selection bias, which is addressed in Supplementary Table S2 and discussed in the Limitations section.

All participants or their legal representatives provided informed consent in the original CLHLS. The CLHLS was approved by the Biomedical Ethics Committee of Peking University. The present study used de-identified publicly available data.

### Assessment of plant-based dietary indices

Dietary intake was assessed at baseline using a food frequency questionnaire (FFQ) administered during face-to-face interviews. Based on the available CLHLS FFQ items and established scoring methods (12), 16 food items were classified into three mutually exclusive groups: (1) healthy plant foods (8 items: whole grains, vegetable oil, fresh fruits, fresh vegetables, legumes, garlic, nuts, tea); (2) unhealthy plant foods (3 items: refined grains, sugar/sugar-rich foods, salt-preserved vegetables); and (3) animal foods (5 items: animal fat, meat, fish, eggs, dairy products). Most items were reported on a 5-level frequency scale (almost every day; quite often/≥1 time per week; occasionally/≥1 time per month; rarely or never), scored as 1–5 quintiles. Four items were binary (Yes/No): whole grains, refined grains, vegetable oil, and animal fat, scored as 5 (Yes) or 1 (No). The theoretical score range for each index is 16–80 (16 items × 1–5 points). Observed ranges were: PDI 32–68, hPDI 29–65, uPDI 26–73. The complete scoring grid for all 16 items across PDI, hPDI, and uPDI is presented in Supplementary Table S1.

Three dietary indices were constructed. For the PDI (overall plant-based diet index), all 11 plant foods were positively scored (higher consumption = higher score) and all 5 animal foods were reverse-scored (higher consumption = lower score). For the hPDI (healthy plant-based diet index), the 8 healthy plant foods were positively scored, while the 3 unhealthy plant foods and 5 animal foods were reverse-scored. For the uPDI (unhealthy plant-based diet index), the 3 unhealthy plant foods were positively scored, while the 8 healthy plant foods and 5 animal foods were reverse-scored. Each index was calculated as the sum of its 16 component scores, yielding a theoretical range of 16 to 80. These adapted indices have been previously applied and validated in the CLHLS by Chen et al. (12), who examined PDI, hPDI, and uPDI in relation to mortality among the general CLHLS older adult population.

To clarify whether the observed associations were driven by the reverse scoring of animal foods, additional plant-only indices (new PDI and new hPDI) were constructed in sensitivity analyses by summing only the plant-food component scores without animal-food reverse scoring. Furthermore, to distinguish the independent associations of plant vs. animal foods, we simultaneously modeled the healthy plant-food score, unhealthy plant-food score, and animal-food score as continuous variables in a single multivariable model.

### Ascertainment of cardiovascular disease

The cardiovascular disease population was defined according to baseline self-reported physician-diagnosed cardiovascular conditions available in the CLHLS. Specifically, three binary variables (0 = no, 1 = yes) were used: (1) heart disease, (2) coronary heart disease, and (3) cerebrovascular disease/stroke. Participants were classified as having CVD if they responded affirmatively to any of these conditions (In this sample, the coronary heart disease variable was combined with cerebrovascular disease for the primary CVD classification, as the two conditions share common pathophysiological pathways and were jointly assessed in the CLHLS questionnaire for the 2008 wave).

### Ascertainment of mortality

The primary endpoint was all-cause mortality during follow-up. Mortality status and date of death were ascertained by the CLHLS through official death certificates when available, and otherwise through interviews with close relatives or local residential committees. Follow-up continued through 2019 (13).

### Covariates

Baseline covariates were selected *a priori* according to previously published CLHLS studies and their potential associations with dietary patterns and mortality. All covariates were assessed at baseline. Sociodemographic characteristics, lifestyle factors, sleep-related variables, socioeconomic status, and medical history were obtained from standardized baseline questionnaires administered through face-to-face interviews. Anthropometric information was obtained from baseline physical measurements, and BMI was calculated as body weight in kilograms divided by height in meters squared.

Age, BMI, sleep duration, and household income were treated as continuous variables. Household income was presented as median (interquartile range, IQR) and log-transformed (natural log) in Cox regression models to reduce skewness. Sex was classified as male or female. Ethnicity was categorized as Han Chinese or other. Marital status was categorized as married or unmarried. Education was categorized as having received formal education or not. Smoking status was classified as never smoker or current/former smoker. Drinking status was classified as never drinker or current/former drinker. Exercise status was classified as exerciser or non-exerciser. Sleep quality was self-reported as very good, good, so-so, bad, or very bad, and modeled using indicator variables with “good” as the reference category. Occupational history was categorized into five groups, with agricultural/forestry/animal husbandry/fishery workers as the reference. BMI was winsorized at the 1st and 99th percentiles to minimize the influence of extreme values; a confirmatory sensitivity analysis excluding participants with BMI < 15 or > 40 kg/m^2^ was also conducted.

### Statistical analysis

Baseline characteristics were summarized according to PDI quartiles. Continuous variables were presented as mean ± standard deviation and compared using one-way analysis of variance. Categorical variables were presented as counts and percentages and compared using Pearson chi-squared tests.

To address potential selection bias introduced by missing cardiovascular disease diagnostic information, we conducted a supplementary analysis comparing baseline characteristics between participants with complete CVD data and those excluded due to missing CVD information (Supplementary Table S2). Reasons for missing CVD data included proxy respondent interviews, cognitive impairment, and abbreviated questionnaires, as described in the Study Design section.

Cox proportional hazards regression models were used to estimate HRs and 95% CIs for the associations between dietary indices and all-cause mortality. The proportional hazards assumption was tested using Schoenfeld residuals (Grambsch-Therneau test); all dietary indices satisfied the PH assumption (all *P* > 0.05, Supplementary Table S10). Three hierarchical models were fitted: Model 1 (unadjusted); Model 2 (adjusted for age, sex, ethnicity, and BMI); and Model 3 (further adjusted for marital status, education, smoking, drinking, exercise, sleep duration, sleep quality, occupation, household income, diabetes, and cancer). Dietary indices were analyzed as continuous variables (per 1-point increase), per-SD increase, and in quartiles (Q1 as reference). Tests for linear trend across quartiles used the median value within each quartile as an ordinal variable. Survival time was calculated as the interval from the baseline interview date (2008) to the date of death or the end of follow-up (2019), whichever occurred first. Participants who survived beyond the follow-up period or were lost to follow-up were treated as censored observations under the assumption of non-informative censoring.

Restricted cubic spline (RCS) models with four knots (placed at the 5th, 35th, 65th, and 95th percentiles of each dietary index) were used to examine potential non-linear dose-response relationships between plant-based dietary indices and all-cause mortality. The reference value was set at the median of each index. The overall association (P-overall) was assessed by likelihood ratio test comparing the RCS model (3 df) with a null model (no dietary variable), and non-linearity (*P*-non-linear) was assessed by LRT comparing the RCS model (3 df) with a linear model (1 df) (R software, version 4.3.1).

## Results

### Baseline characteristics

A total of 1,526 older adults with cardiovascular disease were included in the analysis, among whom 775 deaths occurred during follow-up. According to PDI quartiles, participants in the highest PDI quartile tended to be younger than those in the lowest quartile (mean age 79.1 vs. 82.0 years; *P* < 0.001). The proportion of Han ethnicity also differed across PDI quartiles (*P* = 0.030). BMI, sex, education, smoking, drinking, regular exercise, sleep duration, sleep quality, and income did not differ substantially across PDI quartiles (Table 1).

**Table 1:** Participants demographics and baseline characteristics.

Characteristic	PDI				*p*-value
	Q1 N = 325	Q2 N = 380	Q3 N = 423	Q4 N = 398	
Age, Mean ± SD	82.0 ± 10.25	82.7 ± 10.04	80.4 ± 10.32	79.1 ± 10.01	< 0.001^1^
BMI, Mean ± SD	22.2 ± 7.93	22.2 ± 4.74	23.3 ± 11.02	23.0 ± 7.36	0.144^1^
Sex, *n* (%)					0.653^2^
Female	178 (54.77%)	207 (54.47%)	229 (54.14%)	202 (50.75%)	
Male	147 (45.23%)	173 (45.53%)	194 (45.86%)	196 (49.25%)	
Ethnic, *n* (%)					0.030^2^
Han	305 (93.85%)	367 (96.58%)	403 (95.27%)	390 (97.99%)	
Other	20 (6.15%)	13 (3.42%)	20 (4.73%)	8 (2.01%)	
Marital, *n* (%)					
Currently married	134 (41.23%)	144 (37.89%)	217 (51.30%)	218 (54.77%)	
Divorced	10 (3.08%)	11 (2.89%)	8 (1.89%)	5 (1.26%)	
Never married	3 (0.92%)	2 (0.53%)	2 (0.47%)	2 (0.50%)	
Widowed	178 (54.77%)	223 (58.68%)	196 (46.34%)	173 (43.47%)	
Education, *n* (%)					0.082^2^
Educated	153 (47.08%)	168 (44.21%)	220 (52.01%)	206 (51.76%)	
No education	172 (52.92%)	212 (55.79%)	203 (47.99%)	192 (48.24%)	
Smoking, *n* (%)	52 (16.00%)	49 (12.89%)	62 (14.66%)	66 (16.58%)	0.494^2^
Drinking, *n* (%)	35 (10.77%)	41 (10.79%)	61 (14.42%)	64 (16.08%)	0.070^2^
Exercise, *n* (%)	119 (36.62%)	140 (36.84%)	169 (39.95%)	149 (37.44%)	0.753^2^
Occupation, *n* (%)					
Agriculture, forestry, animal husbandry or fishery worker	192 (59.08%)	211 (55.53%)	231 (54.61%)	237 (59.55%)	
Commercial, service or industrial worker	64 (19.69%)	69 (18.16%)	90 (21.28%)	65 (16.33%)	
Governmental, institutional or managerial personnel	19 (5.85%)	28 (7.37%)	28 (6.62%)	31 (7.79%)	
House worker	19 (5.85%)	26 (6.84%)	23 (5.44%)	21 (5.28%)	
Military personnel	2 (0.62%)	4 (1.05%)	8 (1.89%)	5 (1.26%)	
Never worked	3 (0.92%)	2 (0.53%)	2 (0.47%)	3 (0.75%)	
Others	2 (0.62%)	9 (2.37%)	5 (1.18%)	6 (1.51%)	
Professional and technical personnel	18 (5.54%)	27 (7.11%)	33 (7.80%)	23 (5.78%)	
Self-employed	6 (1.85%)	4 (1.05%)	3 (0.71%)	7 (1.76%)	
Sleep. quality, *n* (%)					0.199^2^
Bad	60 (18.46%)	68 (17.89%)	74 (17.49%)	52 (13.07%)	
Good	117 (36.00%)	123 (32.37%)	137 (32.39%)	165 (41.46%)	
So so	91 (28.00%)	112 (29.47%)	126 (29.79%)	103 (25.88%)	
Very bad	8 (2.46%)	12 (3.16%)	7 (1.65%)	7 (1.76%)	
Very good	49 (15.08%)	65 (17.11%)	79 (18.68%)	71 (17.84%)	
Sleep. hour, Mean ± SD	7.9 ± 3.00	7.6 ± 2.63	7.8 ± 2.83	7.8 ± 2.76	0.641^1^
Income, Mean ± SD	34,208.3 ± 30,673.11	29,337.3 ± 28,944.69	33,246.4 ± 30,161.92	33,691.0 ± 31,611.99	0.118^1^
	25,000 (8,875–50,000)	20,000 (6,000–40,000)	21,800 (9,000–50,000)	20,000 (8,000–50,000)	0.016
Diabetes, n (%)	38 (9.7%)	42 (10.4%)	56 (14.2%)	30 (9.8%)	0.144
Cancer, *n* (%)	6 (1.5%)	7 (1.8%)	5 (1.3%)	7 (2.3%)	0.755
CVD Subtypes, *n* (%)					
Heart disease only	205 (51.0%)	245 (59.3%)	226 (56.8%)	180 (57.5%)	
Stroke only	146 (36.3%)	123 (29.8%)	119 (29.9%)	92 (29.4%)	
Both	51 (12.7%)	45 (10.9%)	53 (13.3%)	41 (13.1%)	0.234

^1^One-way analysis of variance (ANOVA).

^2^Pearson's Chi-squared test.

There are a few extreme BMI values, and a sensitivity analysis will be conducted to verify them.

In the Cox model, when income is treated as a continuous variable, it is logarithmically transformed.

### Associations of plant-based dietary indices with all-cause mortality

In the unadjusted model, higher PDI and hPDI were associated with lower all-cause mortality, whereas higher uPDI was associated with higher mortality. After multivariable adjustment, the association for PDI remained statistically significant. Each 1-point increase in PDI was associated with a 1.4% lower mortality risk (HR = 0.986, 95% CI: 0.973–0.999; *P* = 0.037). Compared with the lowest PDI quartile, the highest quartile was associated with a 21.9% lower mortality risk (HR = 0.781, 95% CI: 0.631–0.966; *P* = 0.023), with a significant trend across quartiles (*P* for trend = 0.020).

hPDI showed an inverse but attenuated association after full adjustment. The continuous hPDI estimate was HR = 0.987 (95% CI: 0.973–1.001; *P* = 0.079), and the highest hPDI quartile was associated with a non-significant lower mortality risk (HR = 0.828, 95% CI: 0.671–1.022; P = 0.079; *P* for trend = 0.061). The proportional hazards assumption was satisfied for hPDI (Schoenfeld residuals test: ρ = −0.070, *P* = 0.051).

In contrast, uPDI was positively associated with all-cause mortality. Each 1-point increase in uPDI was associated with a 1.3% higher mortality risk in the fully adjusted model (HR = 1.013, 95% CI: 1.003–1.024; *P* = 0.009). Compared with the lowest uPDI quartile, the highest quartile showed a higher but borderline association with mortality (HR = 1.235, 95% CI: 0.978–1.558; *P* = 0.076), while the trend across quartiles remained statistically significant (*P* for trend = 0.010) (Table 2). The per-SD estimate was HR = 1.216 (95% CI: 1.007–1.468; *P* = 0.043). The proportional hazards assumption was satisfied for uPDI (Schoenfeld residuals test: ρ = 0.057, *P* = 0.111). The attenuation in the continuous estimate after full covariate adjustment reflects the expanded set of 26 covariates and is consistent with the pattern observed for previously reported PDI–mortality associations; sensitivity analyses using attained age as the time scale and further adjustment for age as a continuous covariate yielded uPDI estimates ranging from *P* = 0.058 to *P* = 0.158, suggesting marginal robustness. While the direction of the uPDI association is consistent with previous literature, the borderline statistical significance in some models warrants cautious interpretation.

**Table 2:** Fully adjusted associations of plant-based dietary indices with all-cause mortality.

Characteristic	Model 1					Model 2			Model 3		
	*N*	Event *N*	HR	95% CI	*p*-value	HR	95% CI	*p*-value	HR	95% CI	*p*-value
PDI (continuous)	1,526	775	0.975	0.963, 0.988	< 0.001	0.984	0.971, 0.996	0.012	0.986	0.973, 0.999	0.037
PDI											
Q1[32, 47]	325	179	—	—		—	—		—	—	
Q2[47, 51]	380	207	0.961	0.786, 1.174	0.697	0.896	0.733, 1.095	0.282	0.889	0.724, 1.093	0.265
Q3[51, 55]	423	198	0.744	0.608, 0.911	0.004	0.81	0.661, 0.992	0.041	0.832	0.675, 1.025	0.084
Q4[55, 68]	398	191	0.724	0.590, 0.888	0.002	0.777	0.633, 0.954	0.016	0.781	0.631, 0.966	0.023
*P* for trend					< 0.001			0.010			0.020
**hPDI (continuous)**	1,526	775	0.976	0.963, 0.990	< 0.001	0.988	0.975, 1.002	0.089	0.987	0.973, 1.001	0.079
hPDI											
Q1[29, 43]	318	192	—	—		—	—		—	—	
Q2[43, 46]	327	170	0.788	0.641, 0.969	0.024	0.850	0.691, 1.046	0.125	0.993	0.803, 1.228	0.947
Q3[46, 50]	480	227	0.812	0.670, 0.985	0.034	0.879	0.725, 1.067	0.192	0.924	0.755, 1.129	0.439
Q4[50, 65]	401	186	0.672	0.549, 0.822	< 0.001	0.821	0.670, 1.007	0.058	0.828	0.671, 1.022	0.079
*P* for trend					< 0.001			0.091			0.061
**uPDI (continuous)**	1,526	775	1.02	1.011, 1.030	< 0.001	1.011	1.001, 1.020	0.032	1.013	1.003, 1.024	0.009
uPDI											
Q1 [26, 45]	317	127	—	—		—	—		—	—	
Q2[45, 51]	429	206	1.116	0.894, 1.393	0.331	0.839	0.669, 1.052	0.128	0.962	0.762, 1.215	0.746
Q3[51, 56]	357	196	1.373	1.098, 1.717	0.005	1.107	0.881, 1.392	0.382	1.167	0.923, 1.476	0.198
Q4[56, 73]	423	246	1.456	1.175, 1.804	< 0.001	1.098	0.881, 1.368	0.406	1.235	0.978, 1.558	0.076
*P* for trend					< 0.001			0.037			0.010

CI, confidence interval; HR, hazard ratio.

Model 1: no covariates were adjusted.

Model 2: adjusted for age, sex, and BMI.

Model 3: adjusted for age, sex, BMI, ethnic, marital, education, cancer, diabetes, drinking, smoking, income, exercise, occupation, sleep hour, and sleep quality.

### Dose-response analyses

**Figure 2:**
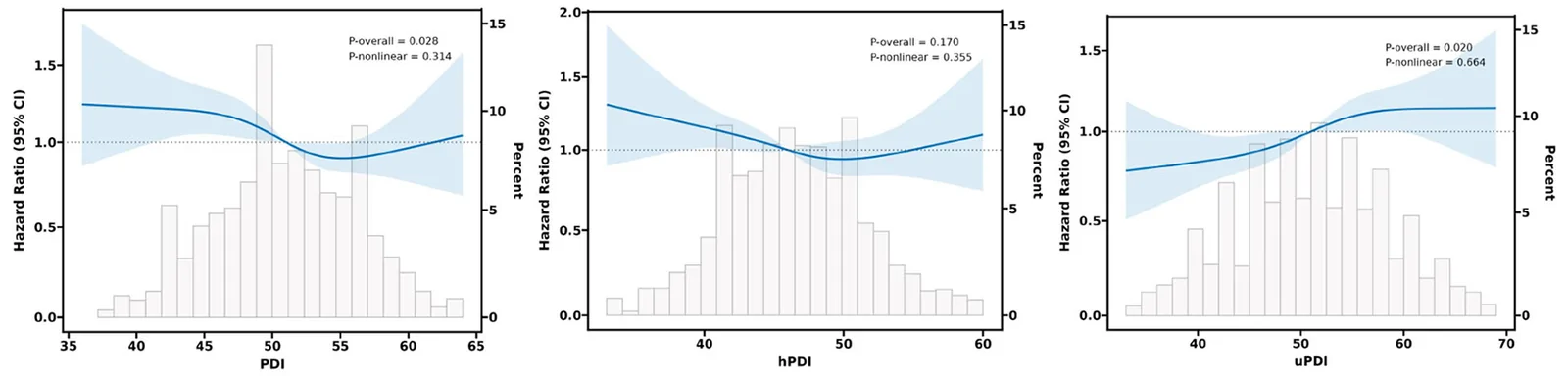
Dose-response analysis of different plant-based dietary indices and all-cause mortality.

Restricted cubic spline analyses showed no statistically significant departure from linearity for any of the three dietary indices (*P*-non-linear: PDI = 0.314, hPDI = 0.355, uPDI = 0.664; *P*-overall from LRT comparing RCS vs. null: PDI = 0.028, hPDI = 0.170, uPDI = 0.020). PDI showed a roughly inverse linear trend across its range (Figure 2), hPDI displayed a shallow inverse slope without clear threshold effects, and uPDI showed a generally positive but shallow slope. The RCS analyses are presented with knot positions marked and the reference set at the median of each index.

Sex-stratified restricted cubic spline analyses showed generally similar directional patterns for PDI, hPDI, and uPDI in men and women (Supplementary Figures S1–S2), although the statistical significance differed across indices and sex groups. No significant non-linear associations were detected. Notably, the spline-based findings were not fully consistent with the sex-stratified Cox subgroup analyses, and the formal tests for interaction by sex were not significant. Therefore, these results should be interpreted cautiously as exploratory supplementary analyses.

### Subgroup analyses

Subgroup analyses showed broadly consistent directions for PDI, hPDI, and uPDI across demographic and lifestyle strata (Supplementary File 1). For PDI, inverse associations were observed in several subgroups, including participants aged >89 years, men, educated participants, non-smokers, non-drinkers, those without regular exercise, and those with good or very good sleep quality. However, no statistically significant interaction was detected across the tested subgroups.

For hPDI, the inverse association was most apparent among participants aged < 76 years and among non-drinkers, but interaction tests were not significant. For uPDI, positive associations were observed in several strata, including older participants, educated participants, non-smokers, and participants reporting regular exercise; again, there was no clear evidence of multiplicative interaction. These findings suggest that the overall pattern of association was not confined to a single subgroup, although subgroup estimates should be interpreted cautiously because of reduced statistical power.

### Sensitivity analyses

The main findings were generally robust across sensitivity analyses (Supplementary File 1). After excluding deaths and censoring events within the first 3 years of follow-up (*n* = 1,395, 770 deaths), PDI remained significantly associated with lower mortality (continuous HR = 0.984, 95% CI: 0.971–0.998; *P* = 0.024), whereas the hPDI association was attenuated (HR = 0.989, 95% CI: 0.976–1.003; *P* = 0.116) and uPDI showed a directionally consistent but non-significant positive association (HR = 1.008, 95% CI: 0.999–1.018; *P* = 0.077).

After excluding participants with baseline diabetes and cancer, PDI remained significantly associated with lower mortality (continuous HR = 0.982, 95% CI: 0.968–0.996; *P* = 0.011; highest vs. lowest quartile HR = 0.774, 95% CI: 0.620–0.966; *P* = 0.024). hPDI remained directionally protective but was not statistically significant after full adjustment, while uPDI remained positively associated as a continuous variable (HR = 1.014, 95% CI: 1.003–1.025; *P* = 0.010).

When dietary scores were standardized, each 1-standard deviation increase in PDI was associated with lower mortality (HR = 0.922, 95% CI: 0.858–0.990; *P* = 0.026), whereas each 1-standard deviation increase in uPDI was associated with higher mortality (HR = 1.087, 95% CI: 1.010–1.171; *P* = 0.026). The standardized hPDI association was attenuated (HR = 0.949, 95% CI: 0.883–1.019; *P* = 0.151).

In plant-only sensitivity analyses (animal-food reverse scoring removed), the new PDI was inversely associated with mortality (continuous HR = 0.982, 95% CI: 0.969–0.995; *P* = 0.008) and the new hPDI showed a directionally inverse association (HR = 0.982, 95% CI: 0.966–0.999; *P* = 0.038). When healthy plant-food, unhealthy plant-food, and animal-food scores were modeled simultaneously, the healthy plant-food score was significantly associated with lower mortality (HR per 1-point increase = 0.977, 95% CI: 0.958–0.997, *P* = 0.027), whereas the animal-food score showed no significant association (HR = 0.994, 95% CI: 0.971–1.018, *P* = 0.619) and the unhealthy plant-food score was not statistically significant (HR = 1.018, 95% CI: 0.997–1.040, *P* = 0.098) (Supplementary Table S11).

## Discussion

In this prospective cohort study of older Chinese adults with established cardiovascular disease, plant-based dietary patterns were differentially associated with all-cause mortality. A higher overall PDI was associated with a lower risk of mortality, whereas a higher uPDI was associated with an increased risk of mortality. The conventional hPDI was associated with a lower, albeit statistically non-significant, risk of mortality after multivariable adjustment. However, when the score was reconstructed using only plant-food components, the plant-food-only hPDI was significantly associated with a lower risk of mortality. These findings suggest that, among older adults with cardiovascular disease, adherence to an overall plant-based dietary pattern and avoidance of unhealthy plant foods may be particularly relevant to survival, whereas interpretation of healthy plant-based scores should account for their scoring structure and the nutritional complexity of older adults with established disease. The present study extends prior work in five key respects. First, unlike previous CLHLS-based analyses that examined PDI–mortality associations in the general older adult population (12), our study is restricted exclusively to older adults with established CVD at baseline, addressing a secondary prevention question of direct clinical relevance. Second, while a recent pooled multi-cohort analysis (9) included CLHLS participants with cardiometabolic conditions, the CLHLS CVD subgroup was not analyzed in isolation with dedicated sensitivity analyses tailored to the CLHLS dietary assessment characteristics (e.g., preserved vegetables, tea, garlic). Third, to our knowledge, this is the first application of plant-only sensitivity indices in a CLHLS CVD population, isolating the association of plant foods themselves by removing animal-food reverse scoring. Fourth, per-SD standardized analyses enable direct comparison of effect magnitudes across PDI, hPDI, and uPDI on a common scale. Fifth, we systematically evaluated the proportional hazards assumption, non-linearity via restricted cubic splines, and the robustness of findings to alternative time scales (attained age), BMI outlier exclusion, and exclusion of early deaths.

### Comparison with previous studies

Our findings are broadly consistent with previous studies showing that the health implications of plant-based diets depend strongly on plant-food quality. In a previous CLHLS study of Chinese older adults, higher PDI and hPDI were associated with lower all-cause mortality, whereas higher uPDI was associated with higher mortality. These findings support the importance of distinguishing healthy from unhealthy plant foods in older Chinese populations (12).

Evidence from cardiovascular disease populations is more limited but particularly relevant to the present study. A recent UK Biobank study among participants with baseline cardiovascular disease reported that higher PDI was associated with lower all-cause mortality, whereas higher uPDI was associated with higher all-cause, cardiovascular, and cancer mortality (14). In contrast, hPDI was not significantly associated with mortality. This pattern is broadly consistent with our results, in which PDI and uPDI showed clearer associations with mortality, while the conventional hPDI was directionally protective but statistically attenuated after multivariable adjustment.

Studies in broader general populations also support the importance of plant-food quality. Previous cohort studies and meta-analyses have generally shown that healthy plant-based dietary patterns are associated with lower all-cause and cardiovascular mortality, whereas unhealthy plant-based dietary patterns are associated with higher mortality risk (15, 16). However, the magnitude and consistency of associations may differ by population, dietary assessment method, baseline disease status, and cause of death (17, 18). Compared with these studies, our analysis focused specifically on older Chinese adults with prevalent cardiovascular disease, a clinically high-risk population with distinct dietary patterns, comorbidity burden, frailty risk, and nutritional requirements.

### Potential explanations and biological mechanisms

The inverse association between PDI and mortality suggests that a more plant-oriented dietary pattern may be associated with improved survival among older adults with cardiovascular disease. This observation is consistent with previous evidence linking plant-based dietary patterns to lower cardiometabolic risk and mortality (6, 19). Plant foods are important dietary sources of dietary fiber, antioxidants, potassium, magnesium, polyphenols, and unsaturated fatty acids. These components may contribute to improvements in blood pressure, lipid metabolism, glycaemic control, endothelial function, gut microbiota composition, and inflammatory status (20–22). For individuals with established cardiovascular disease, these pathways are particularly relevant to long-term prognosis.

Importantly, the present findings also show that plant-based diets are not uniformly beneficial. uPDI was positively associated with mortality, suggesting that a plant-based pattern dominated by refined grains, sugar-rich foods, or preserved plant products may be harmful. In older Chinese adults, refined grains and preserved vegetables may contribute to high glycaemic load and sodium exposure, which can worsen hypertension, vascular injury, kidney stress, and metabolic dysregulation (23–25). Therefore, simply increasing plant-food intake without attention to quality may be insufficient or potentially misleading in dietary guidance for cardiovascular populations.

The attenuated association observed for the conventional hPDI requires careful interpretation. One possible explanation is that the conventional hPDI uses reverse scoring for animal foods. In older adults with cardiovascular disease, a very low intake of animal foods may reflect frailty, poor appetite, post-illness dietary restriction, or inadequate protein intake rather than intentional adherence to a healthy dietary pattern (26, 27). This may attenuate the apparent benefit of healthy plant foods. Another explanation is potential measurement error in food frequency data, given that the CLHLS dietary questionnaire captures consumption frequency rather than detailed portion sizes, nutrient intake, or total energy intake. The plant-food-only sensitivity analysis helps clarify this issue: when reverse scoring for animal foods was removed, the healthy plant-food score was significantly associated with a lower risk of mortality. This pattern supports the biological plausibility of the benefits of healthy plant foods while suggesting that future studies should distinguish plant-food quality from general dietary restriction. With regard to uPDI, the attenuation from Model 3 (HR = 1.013, *P* = 0.009) to Model 2 (HR = 1.011, *P* = 0.032) reflects the comprehensive adjustment for socioeconomic, lifestyle, and health-status covariates. Sensitivity analyses using attained age as the time scale yielded consistent directional estimates (*P* range: 0.058–0.158), and the per-SD estimate remained statistically significant (HR = 1.013, *P* = 0.009), providing complementary evidence. The direction of the uPDI association—higher unhealthy plant-based diet scores associated with higher mortality—is consistent with findings from Chen et al. (12) and the broader PDI literature. Nevertheless, the borderline significance in fully adjusted continuous models suggests that the conventional uPDI may have limited discriminating power in this population when the expanded covariate set is accounted for, and this should be considered when interpreting the findings.

Subgroup analyses provided little evidence of effect modification. Although some stratum-specific estimates appeared statistically significant, formal interaction tests were generally not statistically significant. Thus, the findings should not be interpreted as evidence that the associations between plant-based dietary patterns and mortality were confined to selected subgroups. Rather, the overall pattern suggests potential relevance across age, sex, and lifestyle categories, although the modest sample sizes in some subgroups limited precision. In particular, estimates among participants of non-Han ethnicity, with very poor sleep quality, or who smoked were imprecise because of limited numbers.

The robustness analyses increased confidence in the findings. Excluding early deaths and participants with short follow-up helped reduce concerns about reverse causation, as participants with advanced disease may have altered their diet before death. Excluding participants with diabetes or cancer helped address confounding by major comorbidities and disease-related dietary changes. Standardized analyses facilitated comparisons across dietary indices and suggested that PDI and uPDI had clearer associations than the conventional hPDI. The plant-food-only sensitivity analysis further supported the interpretation that healthy plant foods themselves may be associated with lower mortality risk. Together, these analyses suggest that the observed associations were not solely driven by baseline illness, short-term mortality, or the use of a single scoring approach.

### Clinical and public health implications

From a clinical and public health perspective, these results support dietary recommendations that emphasize the quality of plant foods in older adults with cardiovascular disease. The findings do not necessarily support the adoption of strict vegetarianism. Instead, they suggest a pragmatic dietary pattern characterized by higher consumption of fresh vegetables, fruits, legumes, nuts, tea, and other minimally processed plant foods, alongside lower consumption of refined grains, sugar-rich foods, and preserved vegetables. For older adults with CVD, such guidance should also take into account adequate energy and protein intake, frailty risk, chewing ability, socioeconomic constraints, and culturally specific eating patterns.

### Strengths and limitations

This study has several strengths. It used data from a large, community-based cohort of older Chinese adults with long-term mortality follow-up. The analysis distinguished overall, healthy, and unhealthy plant-based dietary patterns, thereby avoiding the oversimplification that all plant-based diets are beneficial. Multiple model specifications, subgroup analyses, and sensitivity analyses were performed, including standardized scores and plant-only scores that directly addressed the structure of the dietary indices.

Several limitations of this study should be acknowledged. First, CVD status was based on self-reported baseline information in the CLHLS, which may have introduced misclassification. Moreover, CVD was broadly defined using three self-reported conditions (heart disease and stroke), and associations may differ across specific CVD subtypes—a heterogeneity we could not examine. Second, dietary intake was assessed using a FFQ that captures consumption frequency rather than quantitative portion sizes or total energy intake. This precludes estimation of absolute nutrient intake and may introduce measurement error, particularly for foods with highly variable portion sizes (e.g., tea, meat). However, frequency-based indices have been shown to rank individuals adequately by dietary quality in large epidemiological studies, and the CLHLS FFQ has demonstrated reasonable validity for capturing habitual dietary patterns in Chinese older adults (28). Third, dietary patterns were measured only at baseline; therefore, dietary changes during follow-up could not be captured. Fourth, despite extensive adjustment for demographic, socioeconomic, lifestyle, and clinical covariates, residual confounding from unmeasured factors (e.g., medication use, disease severity, physical activity intensity) cannot be excluded. The relatively modest R^2^ and C-statistic values in the fully adjusted models further suggest that unmeasured contributors to mortality risk exist, warranting future studies with more comprehensive clinical, behavioral, and biomarker data. Fifth, the outcome was all-cause mortality; cause-specific deaths (e.g., from CVD or cancer) could not be reliably evaluated because complete and systematically adjudicated cause-of-death information was not available for all deceased participants in the analytic sample.

An important additional limitation concerns sample selection. CVD diagnostic information was missing for a substantial proportion of otherwise eligible baseline participants. In supplementary comparisons, participants with missing CVD data differed from those with complete data in several demographic, socioeconomic, lifestyle, sleep-related, and dietary characteristics. Specifically, excluded participants tended to be older, less educated, and more likely to have proxy respondents, suggesting that the analytical sample may represent a somewhat healthier and more cognitively intact subgroup of older adults with prevalent CVD, rather than the full source population. Therefore, the possibility of selection bias cannot be excluded, and this should be considered when generalizing our findings to institutionalized or cognitively impaired older CVD patients. Finally, the observational design precludes causal inference, and our results may not be directly generalizable to younger populations or to populations with substantially different dietary structures.

## Conclusion

Among older Chinese adults with cardiovascular disease, higher adherence to an overall plant-based dietary pattern was associated with lower all-cause mortality, whereas higher adherence to an unhealthy plant-based dietary pattern was associated with higher mortality. Conventional hPDI showed a protective but statistically attenuated association, while plant-only sensitivity analyses suggested that healthy plant foods were associated with lower mortality when animal-food reverse scoring was removed. These findings highlight that plant-based dietary guidance for cardiovascular populations should focus on plant-food quality, not plant-based eating *per se*. Future studies with repeated dietary assessments, quantitative nutrient data, and cause-specific mortality outcomes are needed to validate these findings and clarify the underlying mechanisms.

## Data Availability

The original contributions presented in the study are included in the article/Supplementary material, further inquiries can be directed to the corresponding author.
