# Supplementary material for: Overall and quality-specific plant-based dietary patterns and all-cause mortality in older adults with cardiovascular disease: a prospective cohort study from the CLHLS
Source: Front Nutr. 2026 Sep 9;13:1899346. doi: 10.3389/fnut.2026.1899346 (PMC13599722; doi:10.3389/fnut.2026.1899346)
Supplement: Supplementary file 1 [file Table_1.docx]

**Supplementary Table S1. Scoring grid for plant-based dietary index components.**

| **Food category** | **Food** | **Frequency** | **PDI** | **hPDI** | **uPDI** |
| --- | --- | --- | --- | --- | --- |
| Plant food | Whole grain | Yes | 5 | 5 | 1 |
|  |  | No | 1 | 1 | 5 |
|  | Vegetable oil | Yes | 5 | 5 | 1 |
|  |  | No | 1 | 1 | 5 |
|  | Fresh fruit | Almost everyday | 5 | 5 | 1 |
|  |  | Quite often | 4 | 4 | 2 |
|  |  | Occasionally | 2 | 2 | 4 |
|  |  | Rarely or never | 1 | 1 | 5 |
|  | Fresh vegetable | Almost everyday | 5 | 5 | 1 |
|  |  | Quite often | 4 | 4 | 2 |
|  |  | Occasionally | 2 | 2 | 4 |
|  |  | Rarely or never | 1 | 1 | 5 |
|  | Legume | Almost everyday | 5 | 5 | 1 |
|  |  | ≥1 time/week | 4 | 4 | 2 |
|  |  | ≥1 time/month | 3 | 3 | 3 |
|  |  | Occasionally | 2 | 2 | 4 |
|  |  | Rarely or never | 1 | 1 | 5 |
|  | Garlic | Almost everyday | 5 | 5 | 1 |
|  |  | ≥1 time/week | 4 | 4 | 2 |
|  |  | ≥1 time/month | 3 | 3 | 3 |
|  |  | Occasionally | 2 | 2 | 4 |
|  |  | Rarely or never | 1 | 1 | 5 |
|  | Nut | Almost everyday | 5 | 5 | 1 |
|  |  | ≥1 time/week | 4 | 4 | 2 |
|  |  | ≥1 time/month | 3 | 3 | 3 |
|  |  | Occasionally | 2 | 2 | 4 |
|  |  | Rarely or never | 1 | 1 | 5 |
|  | Tea | Almost everyday | 5 | 5 | 1 |
|  |  | ≥1 time/week | 4 | 4 | 2 |
|  |  | ≥1 time/month | 3 | 3 | 3 |
|  |  | Occasionally | 2 | 2 | 4 |
|  |  | Rarely or never | 1 | 1 | 5 |
|  | Refined grain | Yes | 5 | 1 | 5 |
|  |  | No | 1 | 5 | 1 |
|  | Sugar | Almost everyday | 5 | 1 | 5 |
|  |  | ≥1 time/week | 4 | 2 | 4 |
|  |  | ≥1 time/month | 3 | 3 | 3 |
|  |  | Occasionally | 2 | 4 | 2 |
|  |  | Rarely or never | 1 | 5 | 1 |
|  | Salt-preserved vegetable | Almost everyday | 5 | 1 | 5 |
|  |  | ≥1 time/week | 4 | 2 | 4 |
|  |  | ≥1 time/month | 3 | 3 | 3 |
|  |  | Occasionally | 2 | 4 | 2 |
|  |  | Rarely or never | 1 | 5 | 1 |
| Animal food | Animal fat | Yes | 1 | 1 | 1 |
|  |  | No | 5 | 5 | 5 |
|  | Meat | Almost everyday | 1 | 1 | 1 |
|  |  | ≥1 time/week | 2 | 2 | 2 |
|  |  | ≥1 time/month | 3 | 3 | 3 |
|  |  | Occasionally | 4 | 4 | 4 |
|  |  | Rarely or never | 5 | 5 | 5 |
|  | Fish | Almost everyday | 1 | 1 | 1 |
|  |  | ≥1 time/week | 2 | 2 | 2 |
|  |  | ≥1 time/month | 3 | 3 | 3 |
|  |  | Occasionally | 4 | 4 | 4 |
|  |  | Rarely or never | 5 | 5 | 5 |
|  | Egg | Almost everyday | 1 | 1 | 1 |
|  |  | ≥1 time/week | 2 | 2 | 2 |
|  |  | ≥1 time/month | 3 | 3 | 3 |
|  |  | Occasionally | 4 | 4 | 4 |
|  |  | Rarely or never | 5 | 5 | 5 |
|  | Dairy products | Almost everyday | 1 | 1 | 1 |
|  |  | ≥1 time/week | 2 | 2 | 2 |
|  |  | ≥1 time/month | 3 | 3 | 3 |
|  |  | Occasionally | 4 | 4 | 4 |
|  |  | Rarely or never | 5 | 5 | 5 |
| Note: PDI: Overall Plant-Based Diet Index; hPDI: Healthy Plant-Based Diet Index; uPDI: Unhealthy Plant-Based Diet Index. | | | | | |

Supplementary Table S2. Baseline characteristics according to cardiovascular disease diagnostic information and cardiovascular disease status

| **Characteristic** | **Group** | | | **p-value** |
| --- | --- | --- | --- | --- |
|  | **1  N = 8,584** | **2  N = 6,282** | **3  N = 1,526** |  |
| **Age, Mean ± SD** | 92 ± 10.06 | 84± 11.17 | 81 ± 10.24 | <0.001^1^ |
| **BMI, Mean ± SD** | 19.89 ± 3.384 | 22.12 ± 5.536 | 22.72 ± 8.181 | <0.001^1^ |
| **PDI, Mean ± SD** | 48.9 ± 5.96 | 50.1 ± 5.97 | 50.8 ± 5.51 | <0.001^1^ |
| **Sex, n (%)** |  |  |  | <0.001^2^ |
| female | 5,212 (60.72%) | 3,429 (54.58%) | 816 (53.47%) |  |
| male | 3,372 (39.28%) | 2,853 (45.42%) | 710 (46.53%) |  |
| **Ethnic, n (%)** |  |  |  | <0.001^2^ |
| han | 8,047 (93.74%) | 5,895 (93.84%) | 1,465 (96.00%) |  |
| others | 537 (6.26%) | 387 (6.16%) | 61 (4.00%) |  |
| **Marital, n (%)** |  |  |  | <0.001^2^ |
| currently married and living with spouse | 1,658 (19.32%) | 2,573 (40.96%) | 713 (46.72%) |  |
| divorced | 25 (0.29%) | 12 (0.19%) | 34 (2.23%) |  |
| never married | 86 (1.00%) | 54 (0.86%) | 9 (0.59%) |  |
| separated | 107 (1.25%) | 133 (2.12%) | 0 (0.00%) |  |
| widowed | 6,708 (78.15%) | 3,510 (55.87%) | 770 (50.46%) |  |
| **Education, n (%)** |  |  |  | <0.001^2^ |
| educated | 2,674 (31.15%) | 2,661 (42.36%) | 747 (48.95%) |  |
| no education | 5,910 (68.85%) | 3,621 (57.64%) | 779 (51.05%) |  |
| **Sleep.hours, Mean ± SD** | 8.3 ± 2.67 | 8.0 ± 2.58 | 7.8 ± 2.80 | <0.001^1^ |
| **Smoking, n (%)** | 1,222 (14.24%) | 1,191 (18.96%) | 229 (15.01%) | <0.001^2^ |
| **Drinking, n (%)** | 1,225 (14.27%) | 1,137 (18.10%) | 201 (13.17%) | <0.001^2^ |
| **Exercise, n (%)** | 1,883 (21.94%) | 2,255 (35.90%) | 577 (37.81%) | <0.001^2^ |
| **Occupation, n (%)** |  |  |  | <0.001^2^ |
| agriculture,forestry,animal husbandry or fishery worker | 5,672 (66.08%) | 4,466 (71.09%) | 871 (57.08%) |  |
| commercial, service or industrial worker | 1,065 (12.41%) | 701 (11.16%) | 288 (18.87%) |  |
| governmental, institutional or managerial personnel | 214 (2.49%) | 168 (2.67%) | 106 (6.95%) |  |
| house worker | 901 (10.50%) | 386 (6.14%) | 89 (5.83%) |  |
| military personnel | 49 (0.57%) | 43 (0.68%) | 19 (1.25%) |  |
| never worked | 74 (0.86%) | 32 (0.51%) | 10 (0.66%) |  |
| others | 135 (1.57%) | 120 (1.91%) | 22 (1.44%) |  |
| professional and technical personnel | 300 (3.49%) | 247 (3.93%) | 101 (6.62%) |  |
| self-employed | 174 (2.03%) | 119 (1.89%) | 20 (1.31%) |  |
| **Sleep.quality, n (%)** |  |  |  | <0.001^2^ |
| bad | 719 (8.38%) | 683 (10.87%) | 254 (16.64%) |  |
| good | 4,382 (51.05%) | 2,767 (44.05%) | 542 (35.52%) |  |
| so so | 2,387 (27.81%) | 1,511 (24.05%) | 432 (28.31%) |  |
| very bad | 67 (0.78%) | 73 (1.16%) | 34 (2.23%) |  |
| very good | 1,029 (11.99%) | 1,248 (19.87%) | 264 (17.30%) |  |
| ^1^One-way analysis of variance (ANOVA) | | | | |
| ^2^Pearson's Chi-squared test  Group 1 consists of those without CVD (cardiovascular disease) information. | | | | |

Supplementary Table S3. Subgroup analyses of the association between hPDI and all-cause mortality

| Subgroup | N | Adjusted HR (95% CI)^*^ | P value | P for interaction |
| --- | --- | --- | --- | --- |
| **Overall (hPDI)** | 1526 | 0.987 (0.973-1.001) | 0.079 |  |
| **Age** |  |  |  | 0.263 |
| <76 | 538 | 0.957 (0.925-0.991) | 0.012 |  |
| 76-89 | 641 | 1.001 (0.981-1.022) | 0.908 |  |
| >89 | 347 | 0.981 (0.958-1.005) | 0.115 |  |
| **Sex** |  |  |  | 0.664 |
| female | 816 | 0.991 (0.972-1.010) | 0.363 |  |
| male | 710 | 0.982 (0.962-1.002) | 0.078 |  |
| **Education** |  |  |  | 0.921 |
| educated | 747 | 0.991 (0.970-1.012) | 0.405 |  |
| no education | 779 | 0.985 (0.967-1.004) | 0.120 |  |
| **Ethnic** |  |  |  | 0.829 |
| han | 1465 | 0.988 (0.974-1.002) | 0.085 |  |
| other | 61 | 0.984 (0.907-1.067) | 0.698 |  |
| **Smoking** |  |  |  | 0.845 |
| no | 1297 | 0.987 (0.973-1.002) | 0.093 |  |
| yes | 229 | 0.994 (0.954-1.037) | 0.784 |  |
| **Drinking** |  |  |  | 0.310 |
| no | 1325 | 0.985 (0.970-1.000) | 0.045 |  |
| yes | 201 | 1.006 (0.965-1.049) | 0.767 |  |
| **Exercise** |  |  |  | 0.713 |
| no | 949 | 0.987 (0.970-1.004) | 0.123 |  |
| yes | 577 | 0.992 (0.967-1.017) | 0.533 |  |
| **Sleep.quality** |  |  |  | 0.926 |
| bad | 254 | 0.991 (0.958-1.025) | 0.604 |  |
| good | 542 | 0.984 (0.960-1.009) | 0.209 |  |
| so so | 432 | 0.998 (0.972-1.025) | 0.887 |  |
| very bad | 34 | 0.938 (0.832-1.057) | 0.291 |  |
| very good | 264 | 0.978 (0.942-1.016) | 0.259 |  |
| ^*^Adjusted for age, sex, bmi, ethnic, marital, education, cancer, diabetes, drinking, smoking, income, exercise, occupation, sleep.hour, and sleep.quality | | | | |

Supplementary Table S4. Subgroup analyses of the association between PDI and all-cause mortality

| Subgroup | N | Adjusted HR (95% CI)^*^ | P value | P for interaction |
| --- | --- | --- | --- | --- |
| **Overall (PDI)** | 1526 | 0.986 (0.973-0.999) | 0.037 |  |
| **Age** |  |  |  | 0.408 |
| <76 | 538 | 0.982 (0.954-1.010) | 0.212 |  |
| 76-89 | 641 | 0.994 (0.975-1.013) | 0.529 |  |
| >89 | 347 | 0.975 (0.953-0.997) | 0.024 |  |
| **Sex** |  |  |  | 0.525 |
| female | 816 | 0.987 (0.969-1.005) | 0.15 |  |
| male | 710 | 0.979 (0.962-0.997) | 0.023 |  |
| **Ethnic** |  |  |  | 0.902 |
| han | 1465 | 0.984 (0.971-0.997) | 0.019 |  |
| other | 61 | 0.987 (0.938-1.038) | 0.609 |  |
| **Education** |  |  |  | 0.419 |
| educated | 747 | 0.991 (0.972-1.010) | 0.341 |  |
| no education | 779 | 0.978 (0.961-0.995) | 0.01 |  |
| **Smoking** |  |  |  | 0.638 |
| no | 1297 | 0.983 (0.969-0.996) | 0.012 |  |
| yes | 229 | 0.990 (0.954-1.027) | 0.592 |  |
| **Drinking** |  |  |  | 0.374 |
| no | 1325 | 0.986 (0.973-0.999) | 0.041 |  |
| yes | 201 | 0.967 (0.933-1.003) | 0.073 |  |
| **Exercise** |  |  |  | 0.438 |
| no | 949 | 0.979 (0.964-0.994) | 0.006 |  |
| yes | 577 | 0.990 (0.966-1.014) | 0.41 |  |
| **Sleep.quality** |  |  |  | 0.313 |
| bad | 254 | 1.008 (0.978-1.040) | 0.603 |  |
| good | 542 | 0.977 (0.957-0.997) | 0.027 |  |
| so so | 432 | 0.989 (0.964-1.013) | 0.363 |  |
| very bad | 34 | 0.983 (0.901-1.072) | 0.703 |  |
| very good | 264 | 0.964 (0.932-0.997) | 0.031 |  |
| ^*^Adjusted for age, sex, bmi, ethnic, marital, education, cancer, diabetes, drinking, smoking, income, exercise, occupation, sleep.hour, and sleep.quality | | | | |

Supplementary Table S5. Subgroup analyses of the association between uPDI and all-cause mortality

| Subgroup | N | Adjusted HR (95% CI)^*^ | P value | P for interaction |
| --- | --- | --- | --- | --- |
| **Overall (uPDI)** | 1526 | 1.013 (1.003-1.024) | 0.009 |  |
| **Age** |  |  |  | 0.263 |
| <76 | 538 | 1.013 (0.991-1.035) | 0.262 |  |
| 76-89 | 641 | 1.001 (0.987-1.015) | 0.884 |  |
| >89 | 347 | 1.019 (1.002-1.037) | 0.033 |  |
| **Sex** |  |  |  | 0.92 |
| female | 816 | 1.010 (0.995-1.024) | 0.186 |  |
| male | 710 | 1.010 (0.997-1.023) | 0.144 |  |
| **Ethnic** |  |  |  | 0.511 |
| han | 1465 | 1.010 (1.001-1.020) | 0.036 |  |
| other | 61 | 1.024 (0.954-1.099) | 0.507 |  |
| **Education** |  |  |  | 0.663 |
| educated | 747 | 1.007 (0.991-1.023) | 0.385 |  |
| no education | 779 | 1.012 (0.999-1.025) | 0.066 |  |
| **Smoking** |  |  |  | 0.372 |
| no | 1297 | 1.012 (1.002-1.023) | 0.022 |  |
| yes | 229 | 1.000 (0.973-1.029) | 0.975 |  |
| **Drinking** |  |  |  | 0.377 |
| no | 1325 | 1.009 (0.998-1.019) | 0.104 |  |
| yes | 201 | 1.024 (0.996-1.052) | 0.09 |  |
| **Exercise** |  |  |  | 0.18 |
| no | 949 | 1.004 (0.992-1.016) | 0.497 |  |
| yes | 577 | 1.019 (1.001-1.037) | 0.037 |  |
| **Sleep.quality** |  |  |  | 0.293 |
| bad | 254 | 1.014 (0.991-1.038) | 0.226 |  |
| good | 542 | 1.012 (0.996-1.029) | 0.134 |  |
| so so | 432 | 0.998 (0.980-1.016) | 0.849 |  |
| very bad | 34 | 1.008 (0.950-1.071) | 0.782 |  |
| very good | 264 | 1.026 (0.999-1.054) | 0.061 |  |
| ^*^Adjusted for age, sex, bmi, ethnic, marital, education, cancer, diabetes, drinking, smoking, income, exercise, occupation, sleep.hour, and sleep.quality | | | | |

Supplementary Table S6. Sensitivity analysis after excluding early deaths and participants with short follow-up

| **Characteristic** | **Model 1** | | | | | **Model 2** | | | **Model 3** | | |
| --- | --- | --- | --- | --- | --- | --- | --- | --- | --- | --- | --- |
|  | **N** | **Event N** | **HR** | **95% CI** | **p-value** | **HR** | **95% CI** | **p-value** | **HR** | **95% CI** | **p-value** |
| **PDI** (continuous) | 1,395 | 770 | 0.976 | 0.963, 0.988 | <0.001 | 0.984 | 0.972, 0.997 | 0.015 | 0.986 | 0.973, 0.999 | 0.031 |
| **PDI** |  |  |  |  |  |  |  |  |  |  |  |
| Q1[32,47) | 290 | 178 | — | — |  | — | — |  | — | — |  |
| Q2[47,51) | 339 | 203 | 0.947 | 0.774, 1.159 | 0.599 | 0.882 | 0.721, 1.080 | 0.224 | 0.875 | 0.713, 1.073 | 0.200 |
| Q3[51,55) | 391 | 198 | 0.747 | 0.610, 0.916 | 0.005 | 0.814 | 0.664, 0.997 | 0.047 | 0.837 | 0.681, 1.029 | 0.092 |
| Q4[55,68) | 375 | 191 | 0.727 | 0.593, 0.892 | 0.002 | 0.78 | 0.636, 0.958 | 0.018 | 0.779 | 0.631, 0.962 | 0.02 |
| P for trend |  |  |  |  | <0.001 |  |  | 0.014 |  |  | 0.021 |
|  |  |  |  |  |  |  |  |  |  |  |  |
| **h**PDI (continuous) | 1,395 | 770 | 0.977 | 0.964, 0.991 | 0.001 | 0.989 | 0.976, 1.003 | 0.119 | 0.991 | 0.977, 1.005 | 0.192 |
| **h**PDI |  |  |  |  |  |  |  |  |  |  |  |
| Q1[29,43) | 291 | 188 | — | — |  | — | — |  | — | — |  |
| Q2[43,46) | 305 | 169 | 0.800 | 0.650, 0.985 | 0.035 | 0.863 | 0.700, 1.063 | 0.166 | 0.934 | 0.756, 1.155 | 0.531 |
| Q3[46,50) | 429 | 227 | 0.83 | 0.684, 1.007 | 0.059 | 0.898 | 0.740, 1.091 | 0.280 | 0.918 | 0.753, 1.120 | 0.400 |
| Q4[50,65) | 370 | 186 | 0.685 | 0.559, 0.840 | <0.001 | 0.839 | 0.683, 1.030 | 0.093 | 0.894 | 0.726, 1.100 | 0.289 |
| P for trend |  |  |  |  | <0.001 |  |  | 0.143 |  |  | 0.289 |
|  |  |  |  |  |  |  |  |  |  |  |  |
| **u**PDI (continuous) | 1,395 | 770 | 1.020 | 1.010, 1.029 | <0.001 | 1.014 | 1.005, 1.024 | 0.003 | 1.007 | 0.997, 1.017 | 0.194 |
| **u**PDI |  |  |  |  |  |  |  |  |  |  |  |
| Q1 [26,45) | 293 | 127 | — | — |  | — | — |  | — | — |  |
| Q2[45,51) | 385 | 206 | 1.116 | 0.894, 1.392 | 0.333 | 1.015 | 0.811, 1.270 | 0.897 | 0.886 | 0.702, 1.119 | 0.310 |
| Q3[51,56) | 328 | 192 | 1.345 | 1.075, 1.684 | 0.01 | 1.206 | 0.960, 1.516 | 0.108 | 1.029 | 0.813, 1.303 | 0.809 |
| Q4[56,73) | 389 | 245 | 1.45 | 1.170, 1.797 | <0.001 | 1.28 | 1.028, 1.593 | 0.027 | 1.074 | 0.850, 1.357 | 0.548 |
| P for trend |  |  |  |  | <0.001 |  |  | 0.005 |  |  | 0.159 |
| Abbreviations: CI = Confidence Interval, HR = Hazard Ratio | | | | | | | | | | | |
| Model 1 : no covariates were adjusted | | | | | | | | | | | |
| Model 2 : adjusted for age, sex, and bmi | | | | | | | | | | | |
| Model 3 : adjusted for age, sex, bmi, ethnic, marital, education, cancer, diabetes, drinking, smoking, income, exercise, occupation, sleep.hour, and sleep.quality | | | | | | | | | | | |

Supplementary Table S7. Sensitivity analysis after excluding participants with baseline diabetes or cancer

| **Characteristic** | **Model 1** | | | | | **Model 2** | | | **Model 3** | | |
| --- | --- | --- | --- | --- | --- | --- | --- | --- | --- | --- | --- |
|  | **N** | **Event N** | **HR** | **95% CI** | **p-value** | **HR** | **95% CI** | **p-value** | **HR** | **95% CI** | **p-value** |
| **PDI (continuous)** | 1,339 | 679 | 0.972 | 0.959, 0.985 | <0.001 | 0.979 | 0.965, 0.992 | 0.002 | 0.982 | 0.968, 0.996 | 0.011 |
| **PDI** |  |  |  |  |  |  |  |  |  |  |  |
| Q1[32,47) | 290 | 162 | — | — |  | — | — |  | — | — |  |
| Q2[47,51) | 340 | 191 | 0.971 | 0.788, 1.198 | 0.786 | 0.890 | 0.722, 1.098 | 0.278 | 0.893 | 0.722, 1.104 | 0.295 |
| Q3[51,55) | 357 | 160 | 0.699 | 0.562, 0.870 | 0.001 | 0.728 | 0.584, 0.906 | 0.005 | 0.758 | 0.606, 0.948 | 0.015 |
| Q4[55,68) | 352 | 166 | 0.697 | 0.561, 0.866 | 0.001 | 0.757 | 0.609, 0.941 | 0.012 | 0.774 | 0.620, 0.966 | 0.024 |
| P for trend |  |  |  |  | <0.001 |  |  | 0.003 |  |  | 0.010 |
|  |  |  |  |  |  |  |  |  |  |  |  |
| **hPDI (continuous)** | 1,339 | 679 | 0.974 | 0.960, 0.989 | <0.001 | 0.984 | 0.969, 0.999 | 0.032 | 0.988 | 0.973, 1.004 | 0.138 |
| **hPDI** |  |  |  |  |  |  |  |  |  |  |  |
| Q1[29,43) | 264 | 171 | — | — |  | — | — |  | — | — |  |
| Q2[43,46) | 290 | 152 | 0.771 | 0.619, 0.960 | 0.020 | 0.833 | 0.669, 1.037 | 0.103 | 0.897 | 0.718, 1.121 | 0.338 |
| Q3[46,50) | 426 | 204 | 0.816 | 0.666, 1.000 | 0.050 | 0.873 | 0.712, 1.071 | 0.193 | 0.906 | 0.735, 1.115 | 0.351 |
| Q4[50,65) | 339 | 152 | 0.645 | 0.518, 0.803 | <0.001 | 0.782 | 0.627, 0.976 | 0.029 | 0.864 | 0.691, 1.080 | 0.200 |
| P for trend |  |  |  |  | <0.001 |  |  | 0.054 |  |  | 0.230 |
|  |  |  |  |  |  |  |  |  |  |  |  |
| **uPDI (continuous)** | 1,339 | 679 | 1.023 | 1.013, 1.034 | <0.001 | 1.014 | 1.003, 1.024 | 0.011 | 1.014 | 1.003, 1.025 | 0.01 |
| **uPDI** |  |  |  |  |  |  |  |  |  |  |  |
| Q1 [26,45) | 315 | 123 | — | — |  | — | — |  | — | — |  |
| Q2[45,51) | 312 | 155 | 1.166 | 0.920, 1.479 | 0.204 | 0.928 | 0.730, 1.181 | 0.544 | 0.906 | 0.710, 1.156 | 0.426 |
| Q3[51,56) | 318 | 171 | 1.349 | 1.069, 1.701 | 0.012 | 1.185 | 0.937, 1.500 | 0.157 | 1.175 | 0.928, 1.488 | 0.18 |
| Q4[56,73) | 394 | 230 | 1.541 | 1.238, 1.919 | <0.001 | 1.242 | 0.993, 1.550 | 0.057 | 1.236 | 0.989, 1.545 | 0.063 |
| P for trend |  |  |  |  | <0.001 |  |  | 0.007 |  |  | 0.006 |
| Abbreviations: CI = Confidence Interval, HR = Hazard Ratio | | | | | | | | | | | |
| Model 1 : no covariates were adjusted | | | | | | | | | | | |
| Model 2 : adjusted for age, sex, and bmi | | | | | | | | | | | |
| Model 3 : adjusted for age, sex, bmi, ethnic, marital, education, cancer, diabetes, drinking, smoking, income, exercise, occupation, sleep.hour, and sleep.quality | | | | | | | | | | | |

Supplementary Table S8. Standardized associations of PDI, hPDI, and uPDI with all-cause mortality

| **Characteristic** | **Model 1** | | | | | **Model 2** | | | **Model 3** | | |
| --- | --- | --- | --- | --- | --- | --- | --- | --- | --- | --- | --- |
|  | **N** | **Event N** | **HR** | **95% CI** | **p-value** | **HR** | **95% CI** | **p-value** | **HR** | **95% CI** | **p-value** |
| **PDI**  **(continuous)** | 1,526 | 775 | 0.975 | 0.963, 0.988 | <0.001 | 0.984 | 0.971, 0.996 | 0.012 | 0.986 | 0.973, 0.999 | 0.037 |
| **PDI**  **(standardized)** |  |  | 0.871 | 0.813, 0.934 | <0.001 | 0.914 | 0.852, 0.980 | 0.012 | 0.922 | 0.858, 0.990 | 0.026 |
| L |  |  | — | — |  | — | — |  | — | — |  |
| M |  |  | 0.891 | 0.747, 1.063 | 0.200 | 0.876 | 0.734, 1.046 | 0.143 | 0.898 | 0.749, 1.076 | 0.242 |
| H |  |  | 0.714 | 0.604, 0.844 | <0.001 | 0.775 | 0.655, 0.916 | 0.003 | 0.796 | 0.671, 0.946 | 0.009 |
| P for trend |  |  |  |  | <0.001 |  |  | 0.003 |  |  | 0.009 |
|  |  |  |  |  |  |  |  |  |  |  |  |
| **hPDI**  **(continuous)** | 1,526 | 775 | 0.976 | 0.963, 0.990 | <0.001 | 0.988 | 0.975, 1.002 | 0.089 | 0.987 | 0.973, 1.001 | 0.079 |
| **hPDI (standardized)** |  |  | 0.884 | 0.824, 0.949 | <0.001 | 0.941 | 0.877, 1.009 | 0.089 | 0.949 | 0.883, 1.019 | 0.151 |
| L |  |  | — | — |  | — | — |  | — | — |  |
| M |  |  | 0.822 | 0.687, 0.983 | 0.031 | 0.887 | 0.741, 1.061 | 0.19 | 0.934 | 0.778, 1.121 | 0.462 |
| H |  |  | 0.776 | 0.656, 0.919 | 0.003 | 0.858 | 0.725, 1.017 | 0.077 | 0.885 | 0.745, 1.051 | 0.162 |
| P for trend |  |  |  |  | 0.004 |  |  | 0.083 |  |  | 0.164 |
|  |  |  |  |  |  |  |  |  |  |  |  |
| **uPDI**  **(continuous)** | 1,526 | 775 | 1.020 | 1.011, 1.030 | <0.001 | 1.011 | 1.001, 1.020 | 0.032 | 1.013 | 1.003, 1.024 | 0.009 |
| **uPDI (standardized)** |  |  | 1.163 | 1.084, 1.248 | <0.001 | 1.083 | 1.007, 1.166 | 0.032 | 1.087 | 1.010, 1.171 | 0.026 |
| L |  |  | — | — |  | — | — |  | — | — |  |
| M |  |  | 1.278 | 1.059, 1.543 | 0.01 | 1.124 | 0.928, 1.361 | 0.233 | 1.122 | 0.925, 1.360 | 0.242 |
| H |  |  | 1.484 | 1.236, 1.782 | <0.001 | 1.214 | 1.006, 1.466 | 0.043 | 1.216 | 1.007, 1.468 | 0.043 |
| P for trend |  |  |  |  | <0.001 |  |  | 0.043 |  |  | 0.043 |
| Abbreviations: CI = Confidence Interval, HR = Hazard Ratio | | | | | | | | | | | |
| Model 1 : no covariates were adjusted | | | | | | | | | | | |
| Model 2 : adjusted for age, sex, and bmi | | | | | | | | | | | |
| Model 3 : adjusted for age, sex, bmi, ethnic, marital, education, cancer, diabetes, drinking, smoking, income, exercise, occupation, sleep.hour, and sleep.quality | | | | | | | | | | | |

Supplementary Table S9. Associations of plant-food-only dietary indices with all-cause mortality

| **Characteristic** | **Model 1** | | | | | **Model 2** | | | **Model 3** | | |
| --- | --- | --- | --- | --- | --- | --- | --- | --- | --- | --- | --- |
|  | **N** | **Event N** | **HR** | **95% CI** | **p-value** | **HR** | **95% CI** | **p-value** | **HR** | **95% CI** | **p-value** |
| **New PDI (continuous)** | 1,526 | 775 | 0.973 | 0.961, 0.986 | <0.001 | 0.982 | 0.969, 0.995 | 0.007 | 0.982 | 0.969, 0.995 | 0.008 |
| **New PDI** |  |  |  |  |  |  |  |  |  |  |  |
| Q1[20,31) | 336 | 196 | — | — |  | — | — |  | — | — |  |
| Q2[31,35) | 392 | 198 | 0.907 | 0.745, 1.106 | 0.336 | 0.88 | 0.721, 1.073 | 0.207 | 0.875 | 0.717, 1.068 | 0.191 |
| Q3[35,39) | 386 | 210 | 0.935 | 0.769, 1.136 | 0.497 | 0.977 | 0.803, 1.189 | 0.814 | 0.99 | 0.813, 1.206 | 0.922 |
| Q4[39,51) | 412 | 171 | 0.667 | 0.543, 0.819 | <0.001 | 0.747 | 0.608, 0.919 | 0.006 | 0.747 | 0.607, 0.920 | 0.006 |
| P for trend |  |  |  |  | <0.001 |  |  | 0.022 |  |  | 0.026 |
|  |  |  |  |  |  |  |  |  |  |  |  |
| **New hPDI (continuous)** | 1,526 | 775 | 0.964 | 0.948, 0.980 | <0.001 | 0.981 | 0.965, 0.998 | 0.026 | 0.982 | 0.966, 0.999 | 0.038 |
| **New hPDI** |  |  |  |  |  |  |  |  |  |  |  |
| Q1[16,28) | 350 | 214 | — | — |  | — | — |  | — | — |  |
| Q2[28,30) | 293 | 159 | 0.824 | 0.670, 1.012 | 0.065 | 0.848 | 0.690, 1.042 | 0.117 | 0.857 | 0.697, 1.055 | 0.145 |
| Q3[30,33) | 410 | 196 | 0.806 | 0.664, 0.979 | 0.030 | 0.903 | 0.743, 1.097 | 0.304 | 0.912 | 0.749, 1.109 | 0.354 |
| Q4[33,50) | 473 | 206 | 0.665 | 0.549, 0.805 | <0.001 | 0.796 | 0.655, 0.967 | 0.022 | 0.809 | 0.664, 0.984 | 0.034 |
| P for trend |  |  |  |  | <0.001 |  |  | 0.043 |  |  | 0.063 |
| Abbreviations: CI = Confidence Interval, HR = Hazard Ratio | | | | | | | | | | | |
| Model 1 : no covariates were adjusted | | | | | | | | | | | |
| Model 2 : adjusted for age, sex, and bmi | | | | | | | | | | | |
| Model 3 : adjusted for age, sex, bmi, ethnic, marital, education, cancer, diabetes, drinking, smoking, income, exercise, occupation, sleep.hour, and sleep.quality | | | | | | | | | | | |

Supplementary Table S10. Sensitivity analyses for the proportional hazards assumption and age modelling approaches

**Panel A: Schoenfeld residual test for proportional hazards assumption**

| **Dietary Index** | **Spearman ρ** | **χ² (df=1)** | **P value** | **PH Assumption** |
| --- | --- | --- | --- | --- |
| PDI | -0.0455 | 1.61 | 0.2057 | Not violated |
| hPDI | -0.0702 | 3.82 | 0.0506 | Not violated |
| uPDI | 0.0573 | 2.55 | 0.1107 | Not violated |

Note: The Schoenfeld test evaluates whether the hazard ratio for each dietary index is constant over time. Spearman correlation (ρ) between scaled Schoenfeld residuals and survival time is reported. P > 0.05 indicates the proportional hazards assumption is not violated. All models are adjusted for the full set of covariates (Model 3).

**Panel B: Sensitivity analysis — alternative age modelling approaches**

| **Dietary Index** | **HR** | **95% CI (Lower)** | **95% CI (Upper)** | **P value** |
| --- | --- | --- | --- | --- |
| **Primary model (linear age)** |  |  |  |  |
| PDI | 0.9854 | 0.9726 | 0.9983 | 0.027* |
| hPDI | 0.9897 | 0.9760 | 1.0035 | 0.143 |
| uPDI | 1.0097 | 0.9994 | 1.0201 | 0.066 |
| **RCS age (4 knots)** |  |  |  |  |
| PDI | 0.9860 | 0.9732 | 0.9989 | 0.034* |
| hPDI | 0.9904 | 0.9767 | 1.0043 | 0.174 |
| uPDI | 1.0100 | 0.9997 | 1.0205 | 0.058 |
| **Attained age as time scale** |  |  |  |  |
| PDI | 0.9923 | 0.9786 | 1.0053 | 0.229 |
| hPDI | 0.9934 | 0.9792 | 1.0069 | 0.229 |
| uPDI | 1.0073 | 0.9972 | 1.0176 | 0.158 |

Note: Primary model uses linear age as a covariate with time-on-study as the Cox time scale. RCS age model replaces linear age with 3 restricted cubic spline terms for age (knots at 5th, 35th, 65th, and 95th percentiles: 66, 75, 85, and 100 years). Attained-age model uses age as the Cox time scale (entry = age at baseline, exit = age at death or censoring). All models are adjusted for the full set of Model 3 covariates. *P < 0.05, **P < 0.01, ***P < 0.001.

Supplementary Table S11. Simultaneous modelling of healthy plant-food, unhealthy plant-food, and animal-food scores and all-cause mortality

| **Model / Food Component** | **HR (per 1-pt)** | **95% CI (Lower)** | **95% CI (Upper)** | **P value** |
| --- | --- | --- | --- | --- |
| **Model A: Healthy plant + Animal food** |  |  |  |  |
| Healthy plant-food score | 0.9774 | 0.9578 | 0.9974 | 0.027* |
| Animal-food score | 0.9943 | 0.9722 | 1.0169 | 0.619 |
| **Model B: Healthy plant + Unhealthy plant** |  |  |  |  |
| Healthy plant-food score | 0.9796 | 0.9606 | 0.9989 | 0.038* |
| Unhealthy plant-food score | 1.0017 | 0.9693 | 1.0352 | 0.920 |

Note: Both models are adjusted for all Model 3 covariates (age, sex, BMI, education, marital status, smoking, drinking, exercise, diabetes, cancer, income, sleep duration, sleep quality, ethnicity, and occupation). Model A includes healthy plant-food score and animal-food score simultaneously. Model B includes healthy plant-food score and unhealthy plant-food score simultaneously. Healthy plant-food score = vegetable + fruit + bean + nut + tea + garlic (range: 6–30). Unhealthy plant-food score = sugar-sweetened beverages + preserved vegetables (range: 2–10). Animal-food score = meat + fish + egg + dairy (range: 4–20). *P < 0.05, **P < 0.01, ***P < 0.001.

Supplementary Table S12. Restricted cubic spline analysis: P-values for overall association and non-linearity

| **Dietary Index** | **RCS Knots (percentiles)** | **Knot Values** | **P-overall** | **P-nonlinear** |
| --- | --- | --- | --- | --- |
| PDI | 5th, 35th, 65th, 95th | 42, 49, 53, 59 | 0.028 | 0.314 |
| hPDI | 5th, 35th, 65th, 95th | 38, 45, 48, 55 | 0.170 | 0.355 |
| uPDI | 5th, 35th, 65th, 95th | 38, 48, 54, 63 | 0.020 | 0.664 |

Note: P-overall is from a likelihood ratio test comparing the RCS model (3 degrees of freedom) with a null model containing no dietary index terms. P-nonlinear is from a likelihood ratio test comparing the RCS model (3 df) with a linear-only model (1 df). Non-significant P-nonlinear indicates no significant departure from linearity. All models adjusted for Model 3 covariates. RCS = restricted cubic spline; df = degrees of freedom.

Supplementary Table S13. Sensitivity analysis excluding participants with extreme BMI values (<1st or >99th percentile)

BMI exclusion criteria: BMI < 14.1 kg/m² (1st percentile) or BMI > 45.0 kg/m² (99th percentile). Total excluded: 32 participants. Analytic sample after exclusion: N = 1494 (events = 755).

| **Dietary Index** | **Sample** | **HR** | **95% CI (Lower)** | **95% CI (Upper)** | **P value** |
| --- | --- | --- | --- | --- | --- |
| PDI | Full sample (N=1526) | 0.9854 | 0.9726 | 0.9983 | 0.027* |
|  | BMI-clean (N=1494) | 0.9855 | 0.9726 | 0.9985 | 0.029* |
| hPDI | Full sample (N=1526) | 0.9897 | 0.9760 | 1.0035 | 0.143 |
|  | BMI-clean (N=1494) | 0.9903 | 0.9765 | 1.0043 | 0.175 |
| uPDI | Full sample (N=1526) | 1.0097 | 0.9994 | 1.0201 | 0.066 |
|  | BMI-clean (N=1494) | 1.0089 | 0.9984 | 1.0195 | 0.095 |

Note: All models are adjusted for Model 3 covariates. Extreme BMI values are defined as values below the 1st percentile or above the 99th percentile of the observed BMI distribution. *P < 0.05, **P < 0.01, ***P < 0.001.

Supplementary Table S14. Distribution of cardiovascular disease subtypes in the analytic sample (N = 1,526)

| **CVD Subtype** | **N** | **%** |
| --- | --- | --- |
| Heart disease (any) | 1046 | 68.5 |
| Heart disease only (no stroke) | 856 | 56.1 |
| Heart disease + Cerebrovascular disease | 190 | 12.5 |
| Cerebrovascular disease (any) | 670 | 43.9 |
| Cerebrovascular disease only (no heart disease) | 480 | 31.5 |

Note: Heart disease is based on self-reported physician diagnosis at baseline (CLHLS question: "Has a physician ever told you that you have heart disease?"). Cerebrovascular disease includes self-reported stroke. The two conditions are not mutually exclusive; 190 participants (12.5%) reported both conditions at baseline.

Supplementary Figure S1：RCS analyses of the associations between PDI, hPDI, uPDI, and all-cause mortality among male participants.

Supplementary Figure S2：RCS analyses of the associations between PDI, hPDI, uPDI, and all-cause mortality among female participants.
